# Cognitive impairment in patients awaiting kidney and liver transplantation—A clinically relevant problem?

**DOI:** 10.1002/brb3.3647

**Published:** 2024-08-12

**Authors:** Aleksandra Golenia, Piotr Olejnik, Magdalena Grusiecka‐Stańczyk, Norbert Żołek, Ewa Wojtaszek, Paweł Żebrowski, Joanna Raszeja‐Wyszomirska, Jolanta Małyszko

**Affiliations:** ^1^ Department of Neurology Medical University of Warsaw Warsaw Poland; ^2^ Medical University of Warsaw Warsaw Poland; ^3^ Department of Hepatology, Transplantology, and Internal Medicine Medical University of Warsaw Warsaw Poland; ^4^ Institute of Fundamental Technological Research Polish Academy of Sciences Warsaw Poland; ^5^ Department of Nephrology, Dialysis and Internal Medicine Medical University of Warsaw Warsaw Poland

**Keywords:** cognitive impairment, end‐stage kidney disease, end‐stage liver disease

## Abstract

**Introduction:**

Cognitive impairment (CI) is common in both end‐stage kidney disease (ESKD) and alcohol‐related liver cirrhosis. The aim of this study was to assess the prevalence and patterns of CI in patients awaiting kidney and liver transplantation, and to identify its determinants.

**Methods:**

In this cross‐sectional, prospective study, 31 consecutive patients with ESKD and 31 consecutive patients with alcohol‐related liver cirrhosis, all currently on transplant waiting lists, were screened for cognitive decline using the Addenbrooke's Cognitive Examination. Medical history, demographics, and laboratory test results were also collected.

**Results:**

The prevalence of CI among patients with ESKD and alcohol‐related liver cirrhosis was 26% and 90%, respectively. In both groups, memory was the most affected cognitive domain, along with verbal fluency in patients with ESKD, and visuospatial abilities in patients with alcoholic cirrhosis. The most statistically significant increase in the prevalence of CI was found in patients with lower educational attainment, in both alcohol‐related liver cirrhosis and ESKD populations as well as in older patients with alcoholic cirrhosis. Furthermore, better cognitive functioning in ESKD patients was associated with higher levels of total lymphocyte count and alanine transaminase (ALT), and in alcohol‐related liver cirrhosis patients with higher levels of ALT and aspartate transaminase. A nonsignificant trend toward lower memory domain scores was also observed with increasing ammonia levels and increasing severity of liver disease (higher Child–Pugh scores). Finally, suboptimal performance on the screening test was correlated with the severity of liver disease as assessed by the Model for End‐Stage Liver Disease Sodium (MELD‐Na), but not at the statistically significant level.

**Conclusions:**

The prevalence of CI, especially in patients with alcohol‐related liver cirrhosis, is high and can be a significant clinical problem, negatively affecting the transplantation process. Routine screening tests in this group would contribute to the implementation of appropriate management, such as rehabilitation program or psychosocial treatments and facilitate the provision of specialized health care.

## Graphical abstract

The prevalence of cognitive impairment among patients with end‐stage kidney disease and alcohol‐related liver cirrhosis awaiting kidney and liver transplantation was 26% and 90%, respectively. In both groups, memory was the most affected cognitive domain, along with verbal fluency in patients with end‐stage kidney disease, and visuospatial abilities in patients with alcoholic cirrhosis.

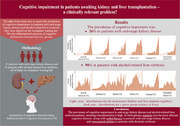

## 1 INTRODUCTION

Cognitive impairment (CI) among patients with end‐stage kidney disease (ESKD) and alcohol‐related liver cirrhosis, awaiting transplantation, remains an underestimated but escalating concern worldwide. Pre‐existing, unrecognized CI may negatively impact the transplantation process by affecting adherence to clinical and pharmacological recommendations (Smith et al., 2017). It is also associated with poorer patient outcomes, including graft rejection (Golfieri et al., 2019). CI in patients with ESKD may be influenced by many traditional and nontraditional cardiovascular risk factors associated with kidney disease (Pépin et al., 2023). Moreover, patients with alcoholic liver disease, but also with other liver disease etiologies such as chronic hepatitis C infection, nonalcoholic fatty liver disease, and primary biliary cirrhosis exhibit an increased susceptibility to developing CI (Butterworth, 1995; Ruck et al., 2023). Excessive alcohol consumption may cause direct neurotoxic effects on the brain, as well as lead to alcoholic liver disease and the build‐up of toxins such as ammonia (Butterworth, 1995). The frontal lobe, the cerebellum, and the limbic system, including the hippocampus, among others, are brain structures particularly vulnerable to alcohol consumption (Oscar‐Berman & Marinković, 2007). Furthermore, alcoholic cirrhosis, which is the final stage of alcoholic liver disease, can affect the structure or functioning of the brain, causing brain damage (Harper, 2009).

Solid organ transplantation is the treatment of choice for both ESKD and alcohol‐related liver cirrhosis (Rai, 2013). In our previous report, we investigated cognitive function, the impact of immunosuppressive therapy and other kidney disease‐related variables on cognitive function in 56 patients after kidney transplantation. We have found that the prevalence of CI in kidney transplant recipients was 30% (Golenia et al., 2023a). As shown in a large, prospective cohort study, the presence of CI in patients with ESKD was associated with a lower chance of being placed on the transplant waiting list, and in patients without diabetes, additionally with increased mortality on the waiting list (Chu et al., 2020). Finally, there are several consistent reports demonstrating the significance of both alcoholic and nonalcoholic fatty liver disease for cognitive decline (Lee et al., 2016; Parikh et al., 2024). Ruck et al. recently published a paper on cognitive dysfunctions in patients with liver disease of various etiologies, indicating that cognitive problems are common in patients with liver disease and are independent of hepatic encephalopathy (Ruck et al., 2023). The prevalence of CI in patients after liver transplantation for liver disease of mixed etiology ranged from 0% to 36% (Siddiqui et al., 2023).

In the present study, we assessed the prevalence of CI in patients with ESKD and alcohol‐related liver cirrhosis who were placed on the transplant waiting list. Due to the different pathological mechanisms of alcoholic and nonalcoholic liver disease, we decided to include only patients with alcoholic liver disease in the study. We also differentiated patterns of cognitive dysfunction between the two groups.

## 2 MATERIALS AND METHODS

Consecutive patients with ESKD and alcohol‐related liver cirrhosis, listed for transplantation, were screened for CI using Addenbrooke's Cognitive Examination (ACE) between January 16, 2023 and May 30, 2023. The protocol for the study was approved by the Bioethics Committee of the Medical University of Warsaw (approval number KB/81/2022). All patients received detailed information regarding the study and researchers obtained written informed consent from every study participant. The research was compliant with the ethical guidelines of the World Medical Association Declaration of Helsinki.

## 3 ESKD PATIENTSˈ RECRUITMENT

Patients were included in the study if they were 18 years of age or older, spoke Polish, had ESKD, were on ambulatory hemodialysis or peritoneal dialysis, and met the hospital's protocol qualifications for kidney transplantation. The following exclusion criteria were used: symptoms of an infectious disease in the previous 8 weeks, and using hypnotics (including benzodiazepines and z‐drugs). Past medical history and laboratory test results were obtained from hospital records.

## 4 ALCOHOL‐RELATED LIVER CIRRHOSIS PATIENTSˈ RECRUITMENT

Patients were included in the study if they were 18 years of age or older, spoke Polish, had alcohol‐related liver cirrhosis (confirmed by radiological imaging and liver biopsy) with features of chronic liver insufficiency, and met the hospital's protocol qualifications for liver transplantation (i.e., no contraindications to this form of therapy were found). Patients were excluded from the study if they had: any infections in the previous 8 weeks or were regularly taking hypnotics (including benzodiazepines and z‐drugs). The stage of liver failure was determined using the Child–Pugh and Model for End‐Stage Liver Disease‐Sodium (MELD‐Na) scores. Past medical history and laboratory test results were obtained from hospital records.

The biochemical parameters were selected on the basis of previously published studies showing their possible association with CI (Gao et al., 2024; Li et al., 2022; Pépin et al., 2023).

## 5 COGNITIVE FUNCTIONS’ ASSESSMENT

The ACE‐III screening test was used to evaluate CI. This test assesses five main cognitive domains, that is, attention, verbal fluency, memory, language, and visuospatial abilities with a score ranging from 0 to 100 points, and takes 15–20 min to complete. Patients were assessed in the morning (between 7:30 a.m. and 11:00 a.m.), in a separate room, in silence, during a scheduled visit to the transplant clinic by one of two researchers. For the diagnosis of mild cognitive impairment (MCI), the range was 88–82 points, and for suspected dementia ≤81 points. At a threshold of 81, the sensitivity for detecting of suspected dementia was 84% and the specificity was 86% (Kaczmarek et al., 2022). The advantages of the ACE‐III test, compared to other cognitive screening tests, lie primarily in: (i) its specificity and sensitivity in CI diagnosis, (ii) its feasibility for administration by doctors and other health care professionals, (iii) the fact that it has been verified using standard neuropsychological tests, (iv) the availability of a validated Polish version, provided at no cost. However, the Polish version of the ACE‐III test for assessing cognitive functions has not been validated for both patients with ESKD and alcohol‐related liver cirrhosis. Nonetheless, it is a validated screening tool assessing global cognitive functioning of elderly people in the general Polish population (Kaczmarek et al., 2022).

## 6 STATISTICAL ANALYSIS

Statistical analysis of the data was performed using IBM SPSS Statistics 26 software. In addition to determining the basic descriptive statistics of the datasets, histograms of the frequencies of test scores in different groups were determined. Besides, receiver operating characteristics (ROC) curves and associated area under the curve (AUC) values were determined to characterize the binary classifier to assess normal group membership based on various parameters collected to characterize patients. A ROC curve is generated by running a logistic regression analysis (Swets, 1996) on a dataset. In the case presented here, binary logistic regression is used to diagnose diseases by assessing the presence or absence of CI based on a patient's test results. There is one binary dependent variable, coded by an indicator variable, where the two values are labeled “0” and “1” (non‐CI and CI), while the independent variable is a continuous variable (test score or diagnostic parameter value). To quantify the basic classification ability of each parameter included as an independent variable, the AUC is calculated. The Student's *t*‐test was used to assess statistical significance when comparing the means of two independent groups.

## 7 RESULTS

### 7.1 Patients characteristics

The study included 31 consecutive patients with ESKD and 31 consecutive patients with alcohol‐related liver cirrhosis, awaiting transplantation. The demographic and clinical characteristics of the study groups are summarized in Table 1. The age distribution was similar in both groups, but in terms of gender distribution, the majority of the alcoholic cirrhosis group were male (77.4%). The mean age for ESKD and alcoholic cirrhosis was 49.94 ± 16.02 years and 55.39 ± 10.48 years, respectively. Furthermore, patients with alcoholic cirrhosis had significantly fewer years of formal schooling than ESKD patients (11.74 ± 4.13 years vs. 15.56 ± 3.76 years, *p* > .001). The mean duration of dialysis in patients with ESKD was 43.45 ± 69.88 months, and patients were on both peritoneal dialysis and hemodialysis (29% vs. 31%).

**TABLE 1:** Demographic characteristics, Addenbrooke's Cognitive Examination‐III (ACE‐III) test results, and blood test results of the study groups.

	Kidney			Liver		
	CI (mean ± SD)	Non‐CI (mean ± SD)	*p*	CI (mean ± SD)	Non‐CI (mean ± SD)	*p*
Age, years	48.63 ± 11.08	46.91 ± 16.78	.038	57.36 ± 9.59	46.33 ± 6.42	.074
Years of education	12.88 ± 2.1	15.83 ± 2.39	.005	11.32 ± 2.11	14.67 ± 4.04	.287
Lymphocytes (10^3^/µL)	1.18 ± 0.37	1.69 ± 0.87	.031	1.28 ± 0.74	0.92 ± 0.25	.110
Hemoglobin (g/dL)	10.48 ± 1.38	11 ± 1.36	.364	11.04 ± 1.58	11.77 ± 2.35	.648
AST (U/L)	19.50 ± 7.91	21.39 ± 8.42	.577	54.50 ± 29.49	126.67 ± 14.15	.002
ALT (U/L)	12.75 ± 4.7	29.43 ± 23.17	.003	36.93 ± 22.69	67.33 ± 21.5	.121
AST/ALT ratio	1.58 ± 0.49	0.91 ± 0.37	.005	1.57 ± 0.54	2.07 ± 0.93	.452
GGTP (U/L)	38.63 ± 43	30.87 ± 19.67	.636	105.18 ± 126.47	109.33 ± 40.77	.905
Creatinine (mg/dL)	8.79 ± 3.45	9.15 ± 2.64	.796	1.085 ± 0.41	0.92 ± 0.14	.194
eGFR (mL/min/1.73 m^2^)	7.12 ± 5.11	5.91 ± 2.83	.540	76.64 ± 26.64	91.33 ± 27.79	.461
Sodium (mmol/L)	139.08 ± 5.04	137.3 ± 3.26	.378	132.44 ± 6.57	132.70 ± 1.67	.871
Potassium (mmol/L)	4.99 ± 1.08	4.65 ± 0.76	.425	4.6 ± 0.7	4.32 ± 0.82	.624
TSH (uIU/mL)	2.53 ± 1.45	2.07 ± 1.24	.445	2.06 ± 1.95	2.13 ± 1.019	.917
Glucose fasting level (mg/dL)	118.75 ± 48.66	101.78 ± 25.23	.372	142.11 ± 81.37	85.33 ± 9.29	.002
Ammonia (µg/d)	–	–	–	95.15 ± 52.2	69.8 ± 17	.011
ACE‐III total score (%)	83.25 ± 5.65	94.43 ± 2.68	.001	68.61 ± 12.9	92.33 ± 0.58	.000
Attention (%)	93.6 ± 6.3	96.09 ± 6.82	.368	82.25 ± 13.7	100.00 ± 0.00	.000
Memory (%)	77.13 ± 7.72	94.04 ± 6.1	.000	57.14 ± 22.62	84.33 ± 6.35	.001
Verbal fluency (%)	73.25 ± 13.81	87.48 ± 7.69	.023	62.21 ± 19.80	90.67 ± 8.08	.004
Language (%)	89.38 ± 10	97.57 ± 3.76	.055	82.07 ± 13.80	96.00 ± 4.00	.003
Visuospatial abilities (%)	80.5 ± 19.96	94.13 ± 8.68	.099	55.43 ± 18.95	91.67 ± 9.71	.006

The *p* values based on *t*‐test of equality of means.

Abbreviations: ALT, alanine transaminase; AST, aspartate aminotransferase; CI, cognitive impairment; eGFR, creatinine levels and creatinine clearance; GGTP, gamma‐glutamyl transferase; non‐CI, noncognitive impairment; TSH, thyroid‐stimulating hormone.

### 7.2 ACE‐III test results

**FIGURE 1:**
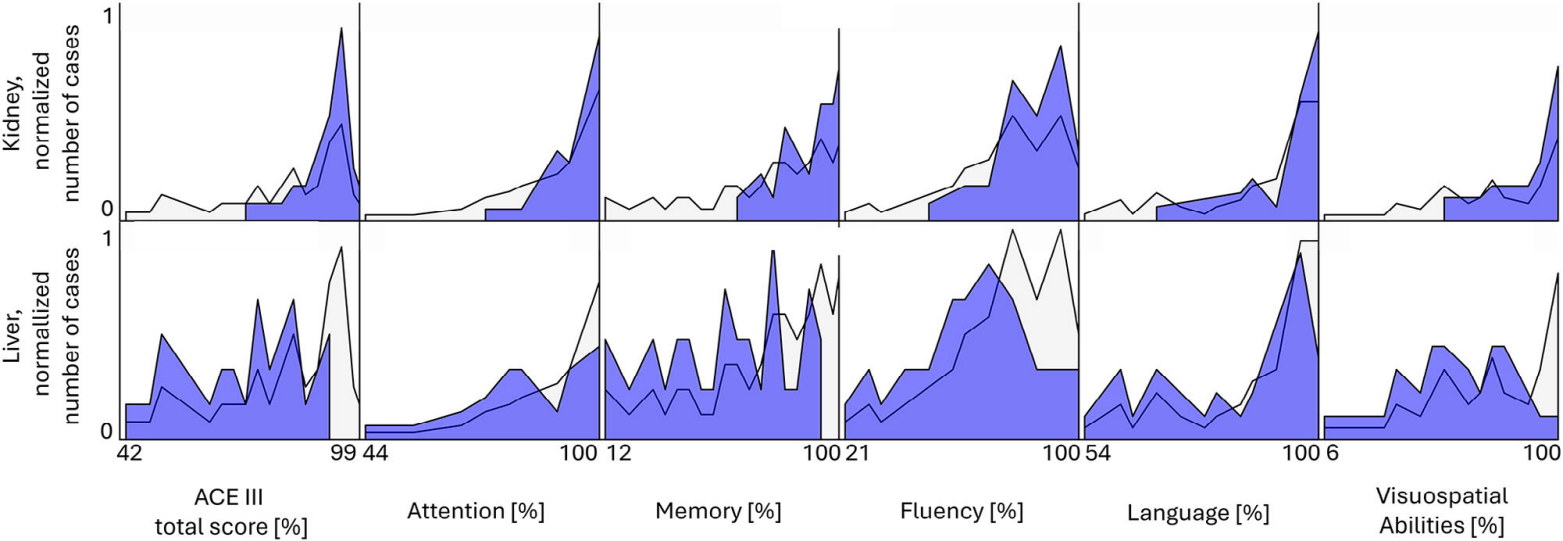
Distributions of normalized scores of Addenbrooke's Cognitive Examination‐III (ACE‐III) test and its individual cognitive domains attention, memory, fluency, language, and visuospatial abilities (columns) in patients with end‐stage kidney disease (ESKD; kidney)—upper row, and alcohol‐related liver cirrhosis (liver)—lower row. Light gray area—distributions for all participants (kidney and liver patients together), dark area—distributions for a given group (kidney or liver). The scale of gray is different in upper and lower row due to normalization.

The ACE‐III test scores were significantly higher in patients with ESKD than in patients with alcoholic cirrhosis (91.58 ± 6.12 vs. 70.9 ± 14.16; *p* = .001, shown in Figure 1). In the group of ESKD patients, the prevalence of CI was 26%, whereas in the group of patients with alcoholic cirrhosis it amounted to 90%. All cognitive domains in patients with alcoholic cirrhosis were significantly impaired as compared to ESKD patients (*p* > .001; Figure 1). The most impaired cognitive domain in both groups was memory, and additionally verbal fluency in patients with ESKD, and visuospatial abilities in patients with alcoholic cirrhosis, as shown in the histograms (*p* > .001, Figure 1).

### 7.3 Factors associated with ACE‐III scores

**FIGURE 2:**
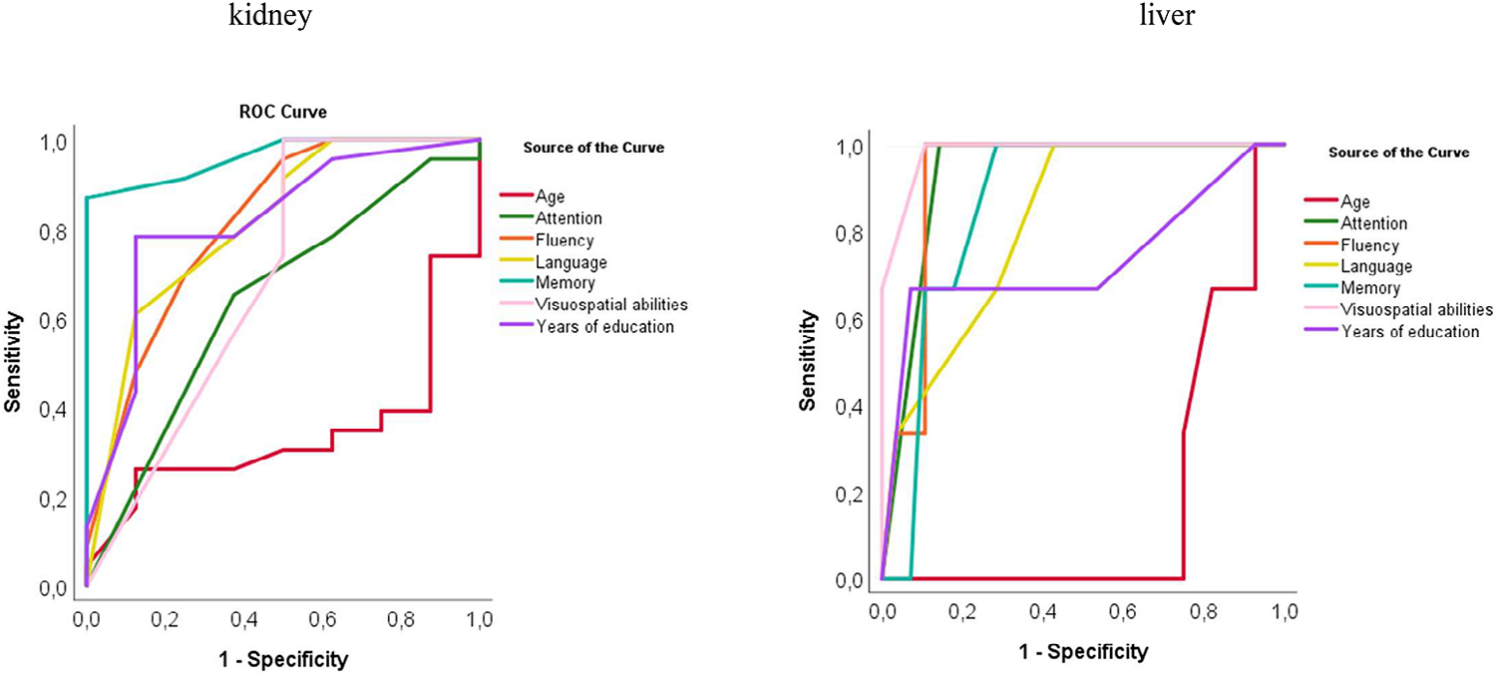
Receiver operating characteristics (ROC) curves for classifying patients without cognitive impairment based on various parameters (age, years of education, total results of the Addenbrooke's Cognitive Examination‐III [ACE‐III] test as well as the results of each domain of the ACE‐III test).

**FIGURE 3:**
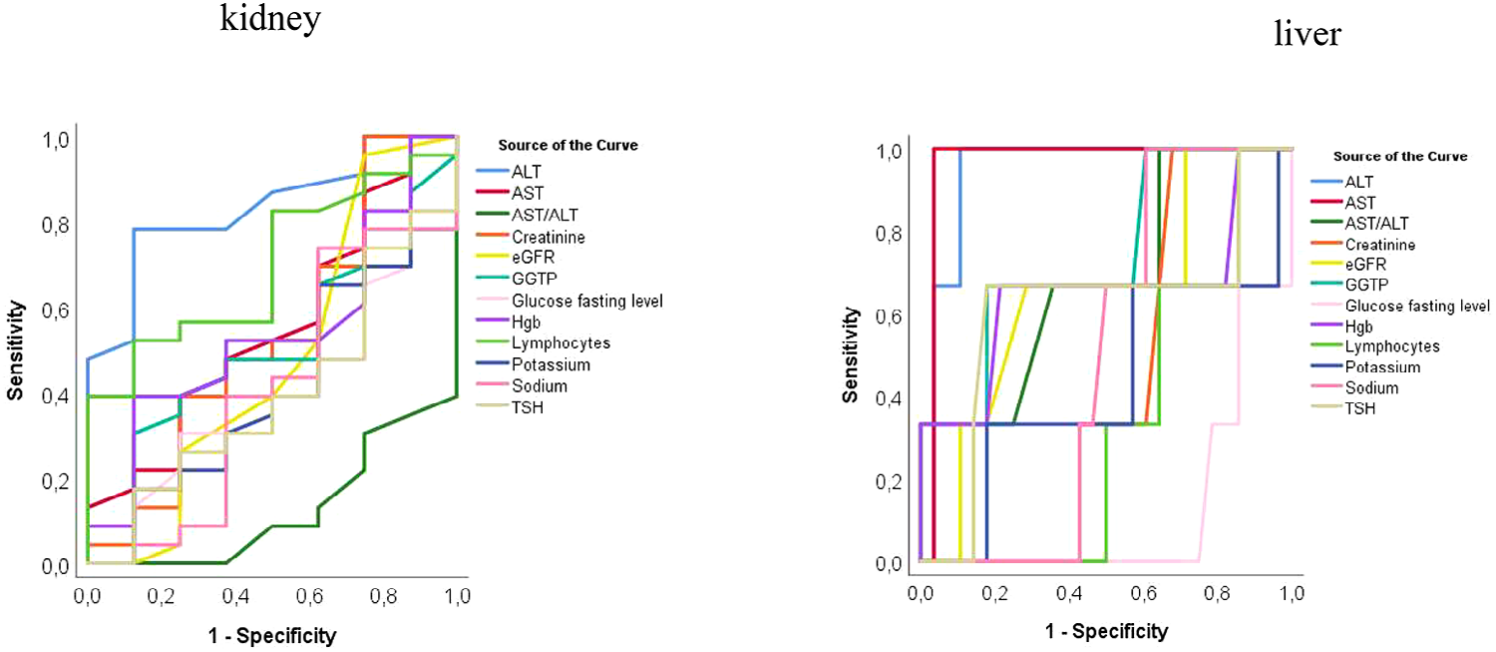
Receiver operating characteristics (ROC) curves for classifying patients without cognitive impairment based on various blood test parameters. Max values of area under the curve (AUC) for ALT (AUC = 0.834) and TLC (AUC = 0.698) in end‐stage kidney disease (ESKD) group, while in alcohol‐related liver cirrhosis group, the best indicators for classification were AST (AUC = 0.964) and ALT (U/L; AUC = 0.940). ALT, alanine transaminase; AST, aspartate aminotransferase; eGFR, creatinine clearance; GGTP, gamma‐glutamyl transferase; TSH, thyroid‐stimulating hormone.

**FIGURE 4:**
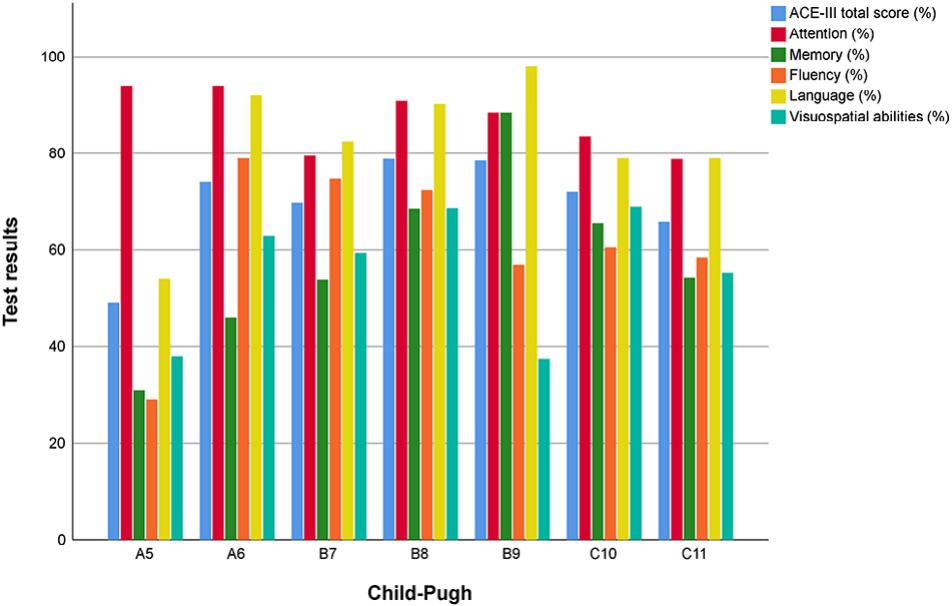
Addenbrooke's Cognitive Examination‐III (ACE‐III) test results and in single cognitive domains depending on the Child–Pugh score. A clearer trend is evident only in the attention domain (red bars), in which the test values decrease slightly as the Child–Pugh score increases. Class A, 5–6 points: well compensated cirrhosis; Class B, 7–9 points: mild decompensated cirrhosis; Class C, 10–15 points: severe decompensated cirrhosis.

**FIGURE 5:**
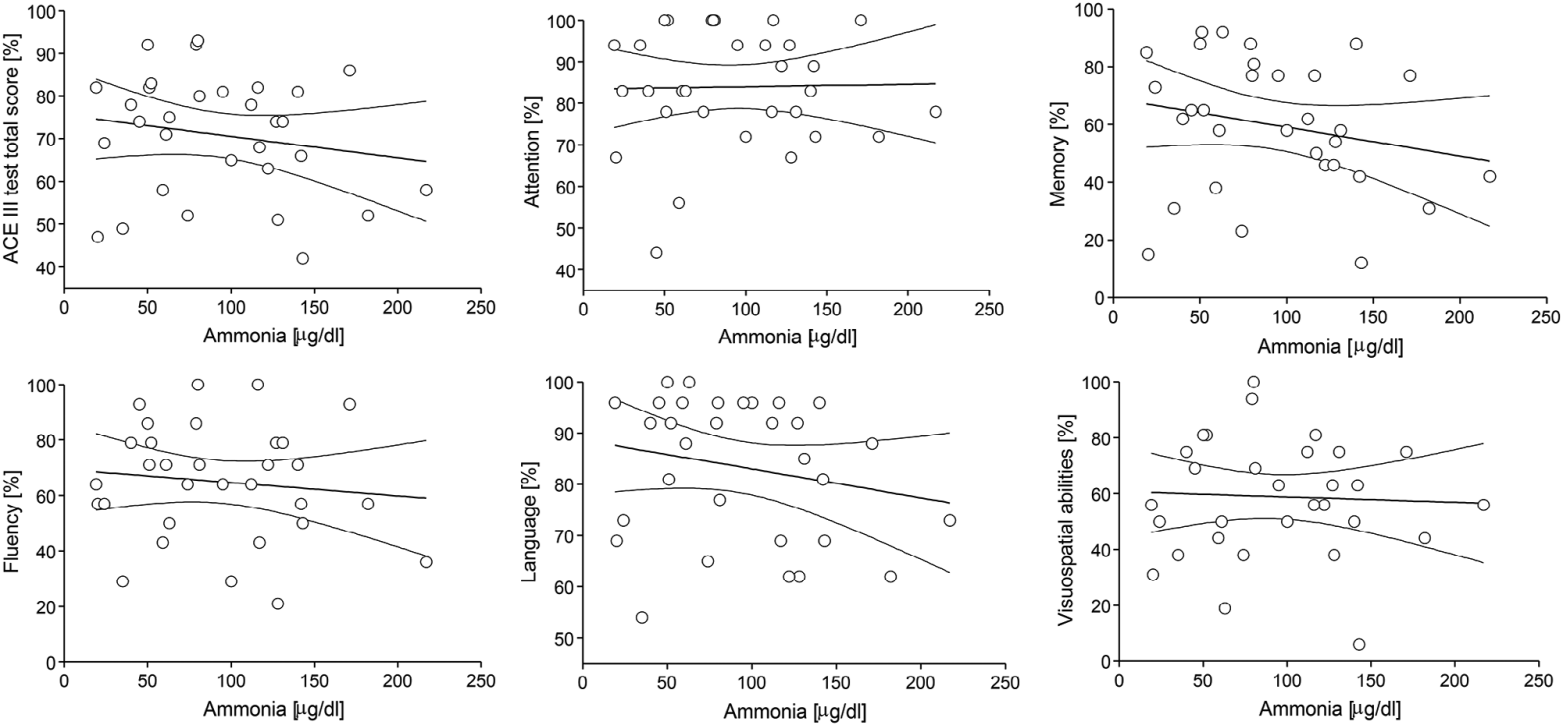
Dependence of total Addenbrooke's Cognitive Examination‐III (ACE‐III) test results and in single cognitive domains attention, memory, fluency, language, and visuospatial abilities on ammonia level in a group of alcohol‐related liver cirrhosis (liver). Linear regression lines (and 95% confidence intervals) indicate a decreasing trend although it is not statistically significant.

**FIGURE 6:**
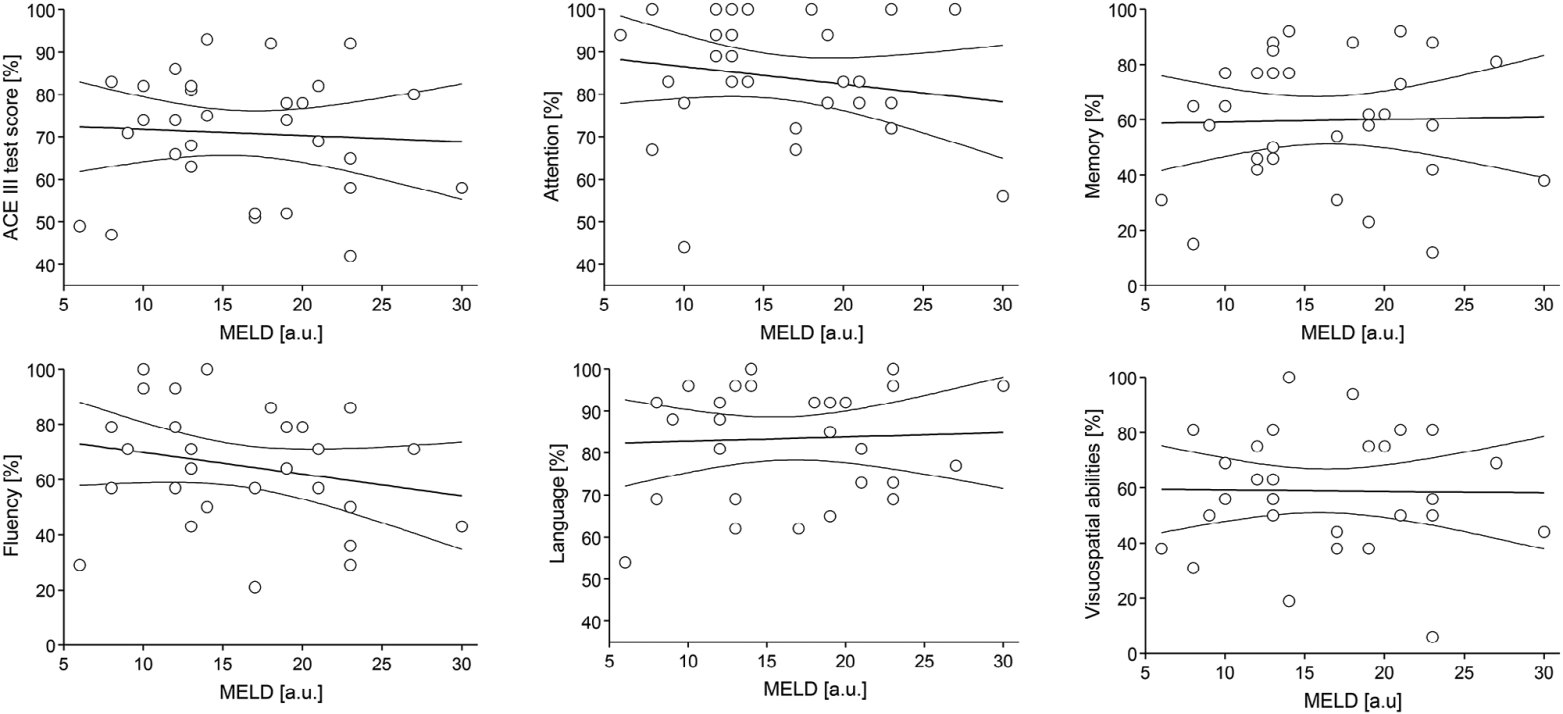
Dependence of the Addenbrooke's Cognitive Examination‐III (ACE‐III) test results and its single domains attention, memory, fluency, language, and visuospatial abilities on the Model for End‐Stage Liver Disease (MELD) scale result in a group of alcohol‐related liver cirrhosis (liver). Linear regression lines (and 95% confidence intervals) indicate a decreasing trend although it is not statistically significant.

The effect of parameter values on their predictive accuracy for binary classification into CI and non‐CI groups was examined. Figure 2 shows ROC curves plotted for age, years of education as well as the results for each domain. With the exception of the attention subdomain and age in the ESKD group, all parameters showed the highest sensitivity and specificity for identifying patients without CI (area under ROC curve AUC at least 0.7; Table 2). In addition, the ROC curves were also plotted for blood test parameters such as aspartate aminotransferase (AST), alanine transaminase (ALT), AST/ALT, gamma‐glutamyl transferase (GGTP), total lymphocyte count (TLC), creatinine levels and creatinine clearance (eGFR), hemoglobin (Hgb) levels, thyroid‐stimulating hormone (TSH), fasting glucose levels, as well as sodium and potassium levels (Figure 3). In the ESKD group, ALT and TLC (maximum values of AUC for ALT was 0.834 and for TLC was 0.698) had the highest sensitivity and specificity for identifying patients without CI, while in the group with alcoholic cirrhosis, AST (AUC = 0.964) and ALT levels (AUC = 0.940) had the best predictive accuracy (Table 3). The other parameters, that is, creatinine levels, creatinine clearance, Hgb levels, TSH, glucose fasting levels, sodium and potassium levels, GGTP, as well as AST in the ESKD group and TLC in the group with alcoholic cirrhosis (the corresponding ROC curves were much closer to the reference line)—had AUC values closer to 0.5, that is, poor predictive accuracy (Table 3). Furthermore, a tendency toward poorer test results in the memory domain was observed with increasing ammonia levels and liver disease severity (higher Child–Pugh scores, Figures 4 and 5). Also, Figure 6 shows the correlation between liver disease severity and cognitive performance. As the MELD‐Na increased, cognitive performance on the ACE‐III test decreased, though this was not statistically significant.

**TABLE 2:** Values of area under the receiver operating characteristics (ROC) curve (AUC) for classification patients without cognitive impairment based on age, years of education, total results of the Addenbrooke's Cognitive Examination‐III (ACE‐III) test, and the results of each domain of the ACE‐III test.

Test result variable(s)	Kidney AUC	Liver AUC
Age, years	0.237	0.179
ACE‐III test	1.000	1.000
Attention	0.625	0.929
Memory	0.962	0.863
Verbal fluency	0.813	0.923
Language	0.810	0.821
Visuospatial abilities	0.700	0.928
Years of education	0.813	0.738

**TABLE 3:** Area under the receiver operating characteristics (ROC) curve (AUC) values for classifying patients without cognitive impairment based on various blood test parameters.

Test result variable(s)	Kidney AUC	Liver AUC
Hemoglobin (g/dL)	0.541	0.655
AST (U/L)	0.554	0.964
ALT (U/L)	0.834	0.94
AST/ALT ratio	0.125	0.685
GGTP (U/L)	0.497	0.696
Creatinine (mg/dL)	0.533	0.429
eGFR (mL/min/1.73 m^2^)	0.478	0.649
Sodium (mmol/L)	0.408	0.494
Potassium (mmol/L)	0.405	0.429
TSH (uIU/mL)	0.397	0.613
Glucose fasting level (mg/dL)	0.394	0.125
TLC (10^3^/µL)	0.698	0.333

Abbreviations: ALT, alanine transaminase; AST, aspartate aminotransferase; eGFR, creatinine clearance; GGTP, gamma‐glutamyl transferase; TLC, total lymphocyte count; TSH, thyroid‐stimulating hormone.

## 8 DISCUSSION

### 8.1 Summary of main results and comparison between other studies

Our study has demonstrated that among the patients awaiting transplantation, the prevalence of CI in patients with alcohol‐related liver cirrhosis was much higher compared to patients with ESKD (90% vs. 26%). In patients with alcohol‐related liver cirrhosis, all cognitive domains were significantly affected, with memory and visuospatial abilities being most impaired. On the other hand, in patients with ESKD, the most impaired domains were memory and verbal fluency, which reflects executive function impairment.

The results of the current study are consistent with the results of previous studies on the association between liver cirrhosis of mixed etiology and cognitive functioning. In a study of 80 patients with compensated liver cirrhosis of alcoholic etiology (*n* = 43) and compensated liver cirrhosis of viral etiology (*n* = 37), more patients with alcoholic compensated liver cirrhosis had impairments in memory and the domain of visuospatial abilities compared with the patients with viral compensated liver cirrhosis (Lee et al., 2016). A previous study of 280 patients with mixed causes of end‐stage liver disease, including 46 patients with alcoholic liver disease, found the most striking impairments to be noted in the domains of attention, immediate memory, and visuospatial construction. Moreover, the prevalence of neuropsychological impairment was highest among patients with liver disease secondary to alcohol abuse (Sorrell et al., 2006).

On the other hand, as we have shown in previously published studies, CI is common in ESKD patients undergoing renal replacement therapy, with prevalence ranging from 30% in kidney transplant recipients, 33% in patients undergoing peritoneal dialysis, to 58% in patients undergoing hemodialysis (Golenia et al., 2023a, 2023b, 2023c), with verbal fluency and memory being the most impaired domains. It should also be noted that CI was found in 55% of a total of 349 dialysis patients evaluated for kidney transplantation, prior to being placed on the active list, and CI was associated with a lower likelihood of being listed for kidney transplantation as well as a longer waiting time for transplantation (Gupta et al., 2019). The median time to active listing for patients with CI was longer compared to patients without CI (10.6 months vs. 6.3 months; Gupta et al., 2019). Similarly, the results of another study showed that patients with ESKD who were cognitively impaired were less likely to be listed, and the median time between dialysis initiation and listing was greater among these patients than in those who did not have CI (11.7 months vs. 4.0 months; Chu et al., 2020).

In our study, the most significant increase in the prevalence of CI was found in patients with lower educational attainment, in both ESKD and alcohol cirrhosis groups, and in elderly patients, but only in the alcohol cirrhosis group. Cognitive abilities are widely recognized to be impacted positively by education levels and negatively by age (Schneeweis et al., 2014). Higher levels of education typically improve cognitive outcomes in several ways, for example, through lifestyle choices, health behaviors, social interactions, labor force participation, type of occupation, and brain development (Schneeweis et al., 2014). Conversely, cognitive functioning declines with normal aging, particularly for cognitive tasks that require the rapid processing or transformation of information to make decisions, for example, processing speed, working memory, and executive cognitive functions (Murman, 2015).

Furthermore, we showed that lower ALT and TLC levels in ESKD patients and lower AST and ALT levels in patients with alcohol cirrhosis negatively affected the ACE‐III test results. If we consider TLC to be a nutritional marker (Leandro‐Merhi et al., 2017), malnutrition may lead to poorer results on cognitive tests. It has also been reported that lower ALT and AST levels are associated with hepatic hypometabolism and result in reduced brain glucose metabolism, impaired neurotransmitter production and synaptic maintenance, systemic insulin resistance and inflammation, and may lead to increased prevalence of dementia (Lu et al., 2021).

We also observed a trend toward poorer performance on cognitive tests in the memory domain as serum ammonia levels and the severity of liver disease increased, as measured by the Child–Pugh score. It is well known that an increase in ammonia levels in the brain can lead to synaptic dysfunction and neurotransmitter imbalance, resulting in memory loss (Jo et al., 2021). Finally, the ACE‐III test results were correlated with disease severity as measured by MELD‐Na score, that is, more advanced disease was associated with poorer cognitive performance.

### 8.2 Possible mechanism contributing to CI in ESKD and alcoholic liver disease

Although the precise mechanisms linking alcoholic cirrhosis and ESKD to CI are not yet well understood, the association may be partly explained. First, alcohol cirrhosis induces neuroinflammation in the brain, notably in the hippocampus, which may contribute to cognitive decline (King et al., 2020). Second, it is well known that brain magnetic resonance imaging (MRI) shows reduced hippocampal volume in patients with alcohol use disorders compared to healthy controls (Zahr & Pfefferbaum, 2017). The hippocampus is involved in memory and learning processes, as well as emotional regulation, by interaction with other brain structures such as the prefrontal cortex, the amygdala, and the nucleus accumbens (Wilson et al., 2017). Third, chronic alcohol abuse may cause damage to the frontal cortex, for example, by increasing the levels of inflammatory cytokines such as tumor necrosis factor alpha (TNFα), interleukin 6 (IL6), monocyte chemoattractant protein 1 (MCP1), and interleukin 1 beta (IL1β) in the prefrontal cortex areas of the brain (King et al., 2020; Zahr & Pfefferbaum, 2017), all responsible for maintaining spatial working memory (van Asselen et al., 2006).

Furthermore, cerebral and glomerular small vessel disease is considered to be one of the causes of cognitive decline in chronic kidney disease (CKD; Mogi & Horiuchi, 2011). Patients with CKD have an increased burden of white matter hyperintensity, silent brain infarction, lacunar infarction, and cerebral microbleeds, which are markers of cerebral small vessel disease and can be detected by brain MRI (Mogi & Horiuchi, 2011). Additionally, there is a well‐documented strong association between cerebral small vessel disease and diminished cognitive functioning in CKD patients (Wei et al., 2022).

Another plausible mechanism that could contribute to CI in CKD patients is the nonvascular hypothesis involving purine nucleotides, oxidative stress, and fibroblast growth factor 23 (FGF23)‐related pathways, as well as the accumulation of uremic toxins, such as phosphate, indoxyl sulfate (IS), and para‐cresyl sulfate (PCS), which are agonists of the transcription factor, that is, the aryl hydrocarbon receptor (AhR), in endothelial cells (Xie et al., 2022). All this may lead to the disruption of the blood–brain barrier and, consequently, increased transport of uremic toxins, inflammatory and procoagulant mediators to the brain, which may lead to the deterioration of cognitive functions (Xie et al., 2022).

Also, oxidative stress, one of the important factors in aging‐related Alzheimer's disease, leads to the cleavage of the amyloid precursor protein (APP) and amyloid beta (Aβ) production/accumulation, accelerating the occurrence of CI (Xie et al., 2022). Additionally, elevated FGF23 levels directly affect hippocampal neurons or disrupt the immune system, impairing memory and learning functions (Xie et al., 2022).

Finally, dysbiosis, which refers to changes in the composition of the gut microbiota, may be another explanation for CI in both ESKD and alcoholic liver disease (Feng et al., 2021). Still, little is known about whether dysbiosis precedes the diseases and serves as an etiological factor, or whether it is merely a comorbid state (Fairfield & Schnabl, 2020; Feng et al., 2021). Among patients with CKD, dysbiosis can affect the course of the disease by increasing proinflammatory cytokines as well as leading to the accumulation of uremic toxins. Both of these factors consequently cause chronic neuroinflammation (Feng et al., 2021; Lee et al., 2023). Moreover, in the course of alcoholic liver disease, the composition of the gut microbiota is constantly changing in parallel with the progression of the disease (Fairfield & Schnabl, 2020). Dysbiosis may be a factor associated with chronic inflammation, although it may serve to deregulate bile acid metabolism, leading to toxin accumulation and subsequent liver failure (Fairfield & Schnabl, 2020). Even though a few studies have highlighted the interrelationship between dysbiosis and CI (Pei et al., 2023), there have been no extensive studies analyzing changes in gut microbiota as a factor affecting cognitive function in both ESKD and alcoholic liver disease.

### 8.3 Limitation

There are several limitations to this study. First, its cross‐sectional design, making it impossible for the results to be used to demonstrate causality. Second, the study groups were relatively small, and the results of our study may be false positives, so they should be treated with caution. Third, a healthy control group was not included in the study. However, to our knowledge, this is the first study in Poland on cognitive function assessment in patients with ESKD and alcohol‐related liver cirrhosis listed for solid organ transplantation. Additionally, the ACE‐III test has a high sensitivity and specificity for detecting CI (Kaczmarek et al., 2022) and is easy to use in outpatient clinics as well as in dialysis units.

The strength of this study lies in the fact that patients with alcoholic cirrhosis included in the study constituted a homogeneous group with the same alcohol‐related etiology of liver disease, which significantly increases the credibility of our results by excluding disease‐related confounding factors that may influence cognitive test results. Homogeneous populations are more often representative than heterogeneous ones, and the more valid conclusions can be drawn even from a small sample (Jager et al., 2017).

## 9 CONCLUSION

The prevalence of CI, especially in patients with alcohol‐related liver cirrhosis, is high and can be a significant clinical problem, negatively affecting the transplantation process. Routine screening tests in this group would contribute to the implementation of appropriate management, such as rehabilitation program or psychosocial treatments and facilitate the provision of specialized health care.

## AUTHOR CONTRIBUTIONS

**Aleksandra Golenia**: Conceptualization; writing—original draft; methodology; writing—review and editing. **Piotr Olejnik**: Investigation; data curation. **Magdalena Grusiecka‐Stańczyk**: Investigation. **Norbert Żołek**: Formal analysis; writing—review and editing. **Ewa Wojtaszek**: Investigation. **Paweł Żebrowski**: Investigation. **Joanna Raszeja‐Wyszomirska**: Supervision; writing—review and editing. **Jolanta Małyszko**: Supervision; conceptualization; writing—review and editing.

## FUNDING INFORMATION

This research received no external funding.

## CONFLICT OF INTEREST STATEMENT

The authors declare no conflicts of interest.

### PEER REVIEW

The peer review history for this article is available at https://publons.com/publon/10.1002/brb3.3647.

## Data Availability

The data that support the findings of this study are available from the corresponding author upon reasonable request.
